# Application of the herbal chemical marker ranking system (Herb MaRS) to the standardization of herbal raw materials: a case study

**DOI:** 10.1186/s12906-023-04178-3

**Published:** 2023-09-30

**Authors:** Bruhan Kaggwa, Godwin Anywar, Edson Ireeta Munanura, Raphael Wangalwa, Henry Kyeyune, Hedmon Okella, Fadhiru Pakoyo Kamba, Ogwang Patrick Engeu

**Affiliations:** 1 Mbarara University of Science and Technology, Pharm-Bio Technology and Traditional Medicine Center (PHARMBIOTRAC), PO Box 1410, Mbarara, Uganda; 2 Department of Pharmacy, Makerere University, College of Health Sciences, P.O. Box 7062, Kampala, Uganda; 3 Department of Plant Sciences, Microbiology & Biotechnology, Makerere University, P.O. Box 7062, Kampala, Uganda; 4 Department of Biology, Faculty of Science, Mbarara University of Science and Technology, P. O BOX 1410, Mbarara, Uganda

**Keywords:** Markers, Phytochemical standardization, Quality control, Herbal materials, Herbal products

## Abstract

**Introduction:**

Phytochemical standardization of herbal materials involves establishing consistent levels of one or more active ingredients or markers. It ensures the authenticity and quality of herbal materials, extracts, and their products. This research aimed to apply the herbal chemical marker ranking system (Herb MaRS) originally proposed for quality assurance of complex herbal products to establish markers for controlling the quality of herbal raw materials.

**Methods:**

The assessment of compounds for suitability as markers was based on the Herb MaRS, with minor modifications as follows: for more objective scoring, evidence of biological activity of the potential marker compound(s) was determined at three levels based on the number of symptoms of the disease condition a compound can treat or alleviate: (i) one symptom (1 point), two symptoms (2 points), and 3 or more symptoms (3 points). The reported concentrations of the compounds were also scored as follows: concentration not determined (0 points), concentration ≥ 5 ppm (1 point), concentration ≥ 50 ppm (2 points) and availability of analytical standards (1 point). Finally, the compounds were scored for the availability of an analytical method (1 point). The compounds were scored from 0 to 8, where 8 indicated the most suitable chemical marker.

**Results:**

The selected markers were as follows: aromadendrine, α-terpineol, globulol, and 1,8-cineol (in *Eucalyptus globulus* Labill. ); aloin, aloe emodin, acemannan (in *Aloe barbadensis* (L.) Burm.f. ), lupeol, lupenone, betulinic acid, betulin, and catechin (in *Albizia coriaria* Oliv.); mangiferin, catechin, quercetin, and gallic acid (in *Mangifera indica* L.); polygodial (in *Warburgia ugandensis* Sprague); azadirachtin, nimbin, nimbidin (in *Azadirachta indica* A. Juss. ); and 6,8,10-gingerols, and 6-shogaol (in *Zingiber officinalis* Roscoe).

**Conclusions:**

Herb MaRS can be efficiently applied to select marker compounds for quality control of herbal materials. However, for herbs whose phytochemicals have not been sufficiently researched, it is difficult to establish evidence of activity, and there are no analytical standards and/or methods; this is the case for plants exclusively used in Africa. The markers identified should be incorporated into chromatographic fingerprints, their quantitative methods developed, and evaluated for applicability at the various stages of the production chain of herbal medicines; then, they can be included in future local plant monographs. There is also a need to build local capacity to isolate marker compounds, particularly those that are not sold by current vendors.

## Introduction

Herbal materials vary greatly in chemical composition due to several factors, including climate, cultivation and harvesting practices, as well as genetic differences among cultivars of the same species [1]. To cater to this variability, herbal raw materials must be standardized before they are used for manufacturing medicinal products. Standardization involves activities that ensure that the materials and the resultant extracts are phytoequivalent. This ensures the reproducibility of the efficacy and safety of the materials and their products [1, 2].

**Fig. 1:**
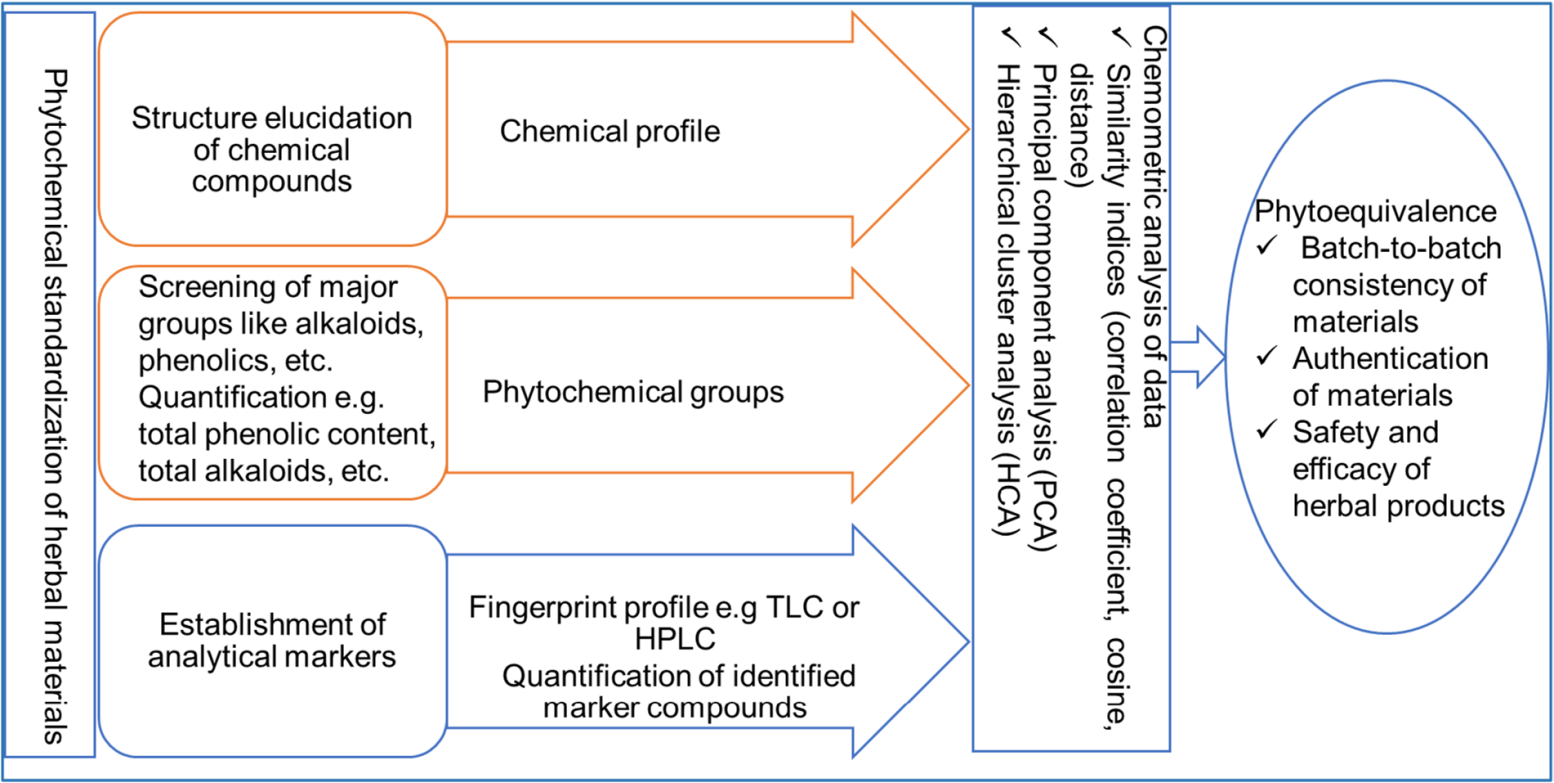
A scheme for phytochemical evaluation of herbal materials

The evaluation of chemical constituents of plant material involves screening and quantification of the major phytochemical groups, the establishment of fingerprint profiles, and/or quantification of selected chemical markers. Once the phytochemical profile is established, the data are evaluated using chemometric methods such as principal component analysis and hierarchical clustering to confirm the phytochemical equivalence of the materials (Fig. 1).

**Fig. 2:**
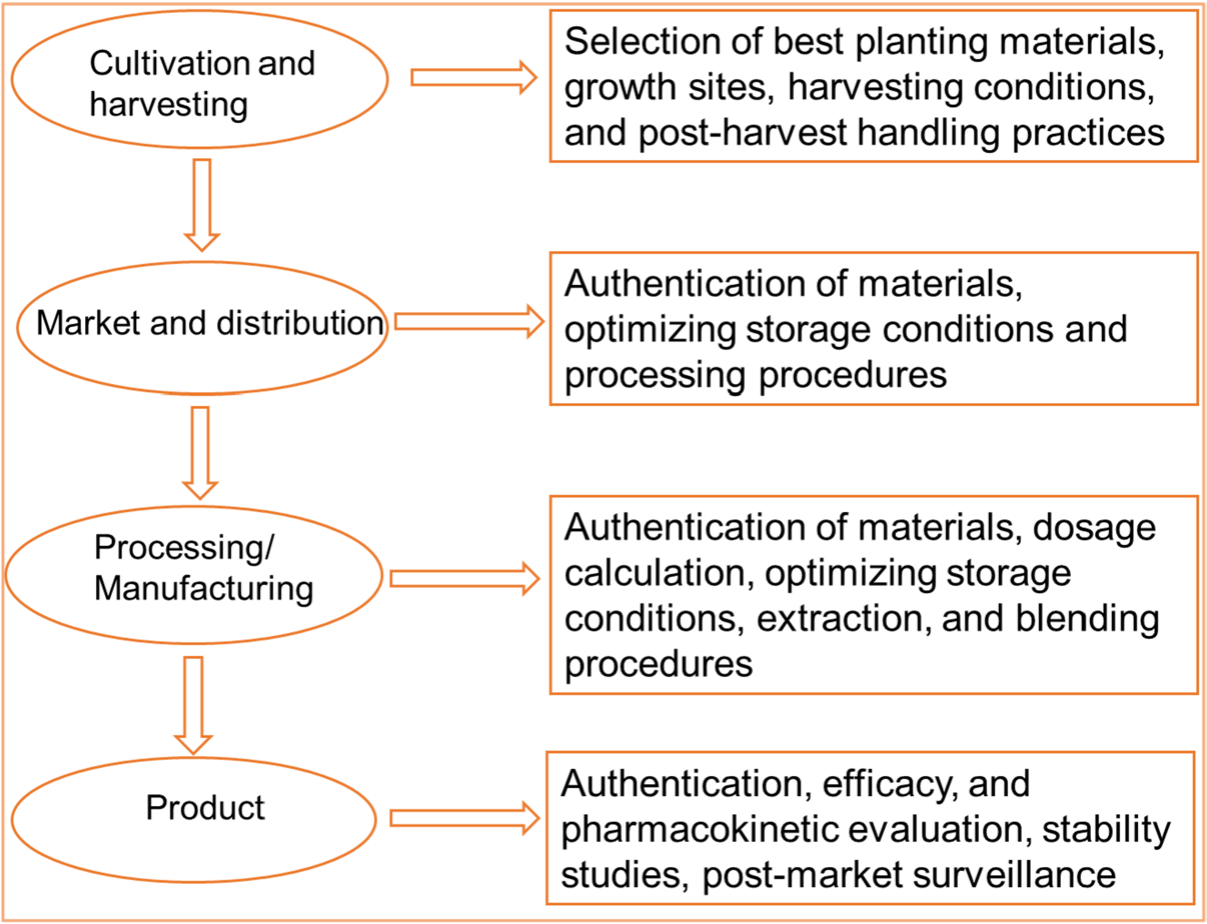
Applications of markers at different stages of the herbal medicine production chain

Markers may be measured in both raw materials and finished products to obtain useful information for various applications. These include the identification and selection of raw materials where concentration limits are set, identification of adulterants and toxicants, assessment of batch-to-batch uniformity of materials from different sources, control of the manufacturing process, assessment of the suitability of packaging and storage, standardization of physiological activities, and calculation of the dosage of raw materials to include in the product formula [2–4] (Fig. 2).

### Selection of marker compounds

When choosing a marker(s) for routine quality control of herbal materials, the following factors should be considered: (i) local availability of effective and easy-to-use analytical methods such thin layer chromatography, high-performance chromatography, and spectroscopy, (ii) availability of analytical standards of acceptable quality, (iii) relevance of the compounds to therapeutic application of the herbal material, and (iv) suitability of the compound(s) as stability indicators. According to the WHO, constituents with known biological activity (related to the traditional use of the herbal material), if known and available, should preferably be selected as markers. Otherwise, compounds with recognized biological activities or characteristic constituents can be used [3]. In line with the WHO and other regulatory guidelines, researchers at the National Institute of Complementary Medicine, University of Western Sydney, proposed a Herbal Chemical Marker Ranking System (Herb MaRS) for selecting markers for quality control of complex herbal products. The Herb MaRS was aimed at providing a uniform and comprehensive guide for the selection of marker compounds for the quality control of polyherbal products. The authors validated this system using an herbal product made from seven herbs. To determine the suitability of phytochemicals as markers, the Herb MaRS ranks the compounds on a scale of 0 to 5. A compound scoring 5 is the most suitable; this is a compound with the highest pharmacological activity related to major symptoms of the disease as claimed by the manufacturer; present in relatively high concentration in the herb or finished product (at least 5 µg/mL); and bioavailable. In addition, it is mandatory to screen toxic compounds, so they are scored 5 by default [5].

In Uganda, comprehensive phytochemical standardization of herbal materials is not yet mandatory since there are no relevant monographs. As such, the products produced from them are not registered; the National Drug Authority issues a “notification” status [6, 7]. For notification, the manufacturer only presents results for general phytochemical screening. Consequently, there have been reports of poor-quality herbal medicines on the market, including adulteration with conventional medicines. The NDA draft guidelines for the regulation of herbal medicines disseminated in 2021, for comments, have recommended the quantification of markers and the establishment of chromatographic fingerprints with reference to the WHO guidelines (https://www.nda.or.ug/wp-content/uploads/2022/03/Guidelines-on-Regulation-of-Traditional-and-Local-Herbal-Medicnes-in-Uganda_Draft-2.pdf). However, since most plants have no monographs yet [8], manufacturers will not know what analytical markers and/or methods to use.

**Fig. 3:**
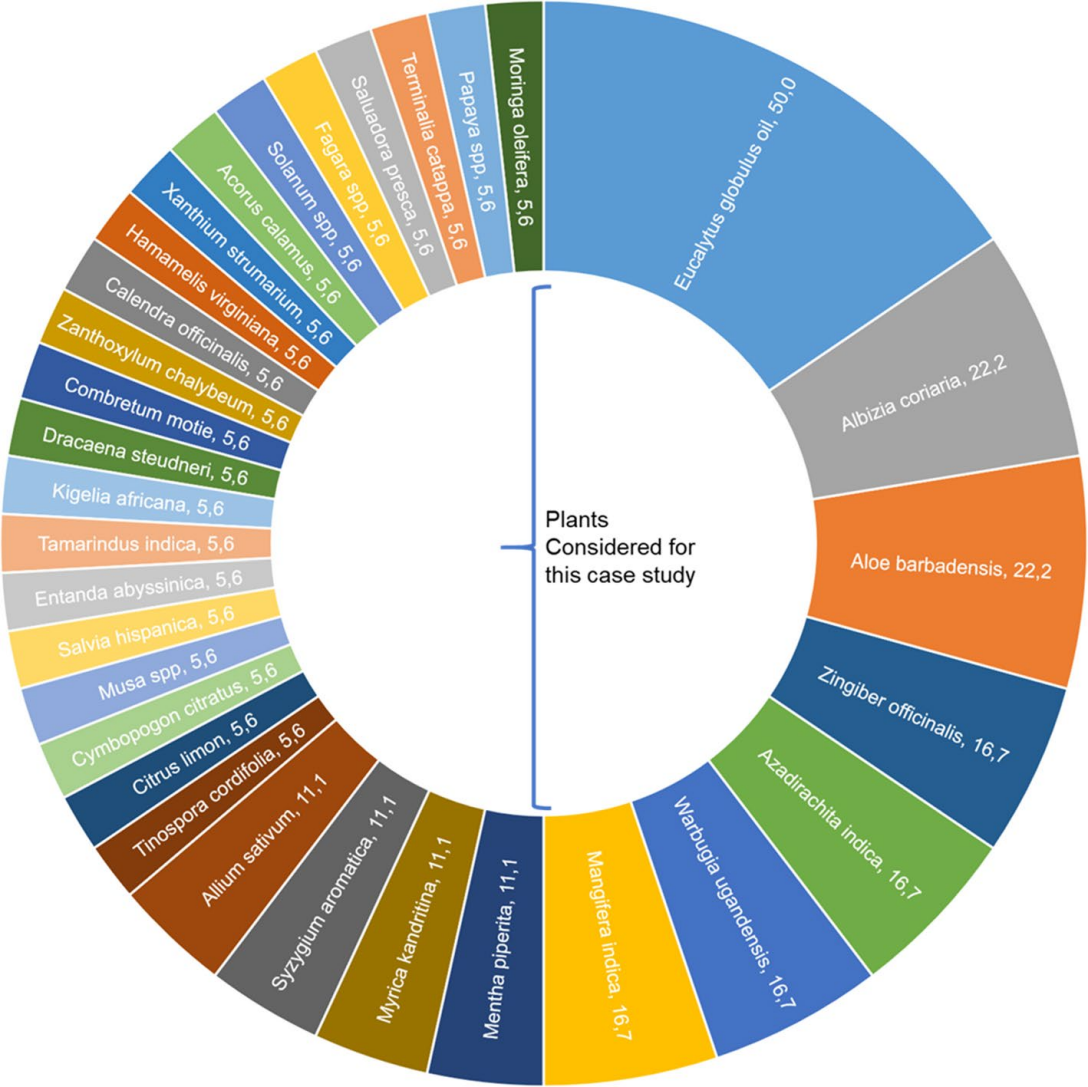
Popularity of herbal materials in Ugandan registered herbal products. The numbers indicate the percentage of products that contain the plant as active ingredient. modified with permission from [7]

The aim of this work was to assess the applicability of the Herb MaRS to establish the quality control of herbal materials. To achieve this, a case study of the seven most commonly used plant species in the manufacture of herbal medicinal products in Uganda was conducted. According to our previous study [7], *Eucalyptus globulus* Labill., *Aloe barbadensis* (L.) Burm.f., *Albizia coriaria* Oliv., Mangifera *indica* L., *Warburgia ugandensis* Sprague, *Azadirachta indica* A. Juss. and *Zingiber officinalis* Roscoe were the most frequently used plants (Fig. 3).

It is evident from Fig. 3 that the seven plant materials considered for this case study are as popular as the other 25 plants combined.

## Methods

### Identification of potential marker compounds

The suitability of analytical markers was determined based on the WHO guidelines for selecting marker substances of herbal origin for quality control of herbal medicines [3] and on the Herbal Chemical Marker Ranking System (Herb MaRS) [5]. Both the WHO guidelines and the Herb MaRS give priority to a compound whose biological activity is related to the traditional use of the plant and can be identified and quantified by the analytical methods available. Additionally, the compound chosen should be available commercially in pure form.

### Establishment of active compounds and evidence of biological activity

An extensive literature search to identify the bioactive compounds and to establish evidence of their biological activity relevant to the therapeutic claims made on the product label was performed for the seven most commonly used herbal materials. Such evidence included studies reporting on the ability of the compound(s) to treat or ameliorate one or more symptoms of the disease condition as indicated by the manufacturer. According to Kaggwa et al. [7], *Albizia coriaria*, *Mangifera indica*, and *Zingiber officinale* are exclusively used in cough syrups; *Warburgia ugandensis* in cough and anti-ulcer syrups; *Eucalyptus globulus* in mouth washes, cough syrups and pain balms; *Aloe vera* in mouth washes, cough syrups, lip balms and GIT cleansing tablets; and *Azadirachta indica* in cough, anti-ulcer syrups and lip balms. The same study established evidence of the efficacy and safety of the plant materials for these therapeutic applications. Table 1 summarizes the diseases (or their symptoms) the products are indicated to manage.

**Table 1:** Disease conditions managed by the most commonly used herbal materials in herbal manufacturing in Uganda. Table modified with permission from [7]

No.	Plant material Source of plant Part of plant used	Dosage form of product containing the material	Indication(s) of the products containing the material
1	*Eucalyptus globulus* Labill. (Myrtaceae) Wild, cultivated Leaf oil	Mouth wash	Toothache, bad odor, sensitivity, bleeding gums, (tooth) cavities, tooth decay, antibacterial, mouth sores
		Cough syrups	Cough, common cold, catarrh, sore throat, congestion from asthma, bronchitis, whooping cough, allergic conditions- sinusitis, rhinitis, mouth sores, hiccups, relieving fever, measles symptoms
		Pain balm	Pain relief
2	*Aloe vera *Burm.f. (Asphodelaceae) Cultivated Whole leaf	Mouth wash	Toothache, bad odor, sensitivity, bleeding gums, (tooth) cavities, tooth decay, antibacterial,
		Aloe tablets	Cleanses GIT
		cough syrup	Cough, flu, sore throat, sinusitis
		Lip balm	Dry, cracked, and painful lips
3	*Albizia coriaria* Oliv. (Fabaceae*)* Wild Stem bark	(Cough) syrups	Whooping cough, catarrh, sore throat, congestion from asthma and bronchitis, relieving fever, sinusitis
4	*Mangifera indica* L. (Anacardiaceae) Wild, cultivated Stembark, leaves		Whooping cough, catarrh, sore throat, congestion from asthma and bronchitis
5	*Warburgia ugandensis* Sprague (Canellaceae) Wild, cultivated Stem bark, leaves		Cough, flu, mouth sores, measles symptoms, common colds, sinusitis, rhinitis, asthma, catarrh, whooping cough, bronchial congestion, mouth sores, hiccups
		(antiulcer) syrups	(GIT) Ulcers
6	*Azadirachta indica* A. Juss. (Meliaceae) Wild, cultivated Stem bark, leaves	Cough syrup	Cough, flu, sore throat, sinusitis
		(antiulcer) syrups	Gastric ulcers, stomach ulcers, flatulence, constipation
		Lip balm	Dry, cracked, and painful lips
7	*Zingiber officinale* Roscoe (Zingiberaceae) Cultivated Rhizome	cough syrups	Allergic cough, smokers cough, whooping cough, productive cough, flu, lung cleaning, sore throat, sinusitis, bronchial asthma, relieving fever

A systematic search for articles was performed using search engines such as Google and indexes including PubMed, Google Scholar, ResearchGate, and Web of Science. The search terms consisted of the chemical name, pharmacological or therapeutic activity of interest, such as “anti-inflammatory activity of 6-gingerol”, plant botanical name and bioactive compounds thereof, such as “bioactive compounds in *Mangifera indica* leaves”, and “mechanism of action of mangiferin”. Only full-length articles published in English were reviewed. We did not limit the search to any timeline since evidence is considered valid until disputed by new findings.

### Availability of analytical methods for the potential marker compounds

In addition to evidence of biological activity of the identified compounds, information regarding quality control methods recommended by existing pharmacopoeial monographs was included, particularly the WHO monographs on selected medicinal plants [9–13], the African Pharmacopoeia, the West African Herbal Pharmacopoeia [14] and the Pharmaceutical monographs for South African plants species [15]. Additionally, the availability of assay methods for the quantification of markers in the respective plant materials was crucial. The primary focus was on high-performance liquid chromatography (HPLC) methods because they are highly sensitive, specific, versatile and readily accessible even in resource-limited countries. Where HPLC methods were not available or not suitable, such as for essential oils, high-performance thin layer chromatography (HPTLC), gas chromatography (GC) or ultraviolet/visible (UV/VIS) spectrophotometric methods, and other available methods were considered. Both HPTLC-densitometry and spectrophotometric methods are less sensitive for the quantitative determination of markers than HPLC, although they are easier to use. On the other hand, GC-based methods are as sensitive as HPLC but are selective to only compounds that are volatile, such as essential oils, while some compounds can be derivatized to make them volatile, the analysis cost is escalated by expensive derivatization reagents.

### Availability of analytical reference standards for the potential marker compounds

Information on the availability of analytical standards and the prices for the smallest units was obtained, preferably from the Sigma Aldrich ® website (https://www.sigmaaldrich.com/UG/en). This was for two main reasons: (i) from our experience, Sigma Aldrich is among the most reliable suppliers of high-quality chemicals, and (ii) they willingly display the prices for various grades and quantities of the same analytical standard. If the compounds were not available from Sigma Aldrich®, a general Google search to establish other potential sources was executed. Finally, the cost of 1 mg or mL of the marker was computed with an assumption that this amount is sufficient for a single analysis to construct calibration curves. We believe that the cost of equivalent grades of standards from other vendors will differ only slightly.

### Selection of the most suitable marker compounds

Using a modified Herb MaRS, compounds were given scores from 0 to 8, where 8 indicates the most suitable chemical marker. The Herb MaRS [5] does not elaborate on how the individual attributes of the compound are scored but rather gives a lumpsum mark after the overall assessment. Therefore, for more objective scoring, we modified the ranking system as follows: evidence of biological activity was divided into three levels based on the number of symptoms of the disease condition a compound can treat or alleviate: (i) one symptom (1 point), two symptoms (2 points), 3 and more symptoms, with well-elucidated mechanisms of action (3 points). We also scored the reported concentrations of the compounds in the plant material (concentration not determined (0 points), concentration ≥ 5 ppm (1 point), (concentration ≥ 50 ppm, 2 points) and availability of analytical standards (1 point); last, we scored the availability of an analytical method (1 point).

## Results

### Evidence of biological activity of potential chemical markers

**Fig. 4:**
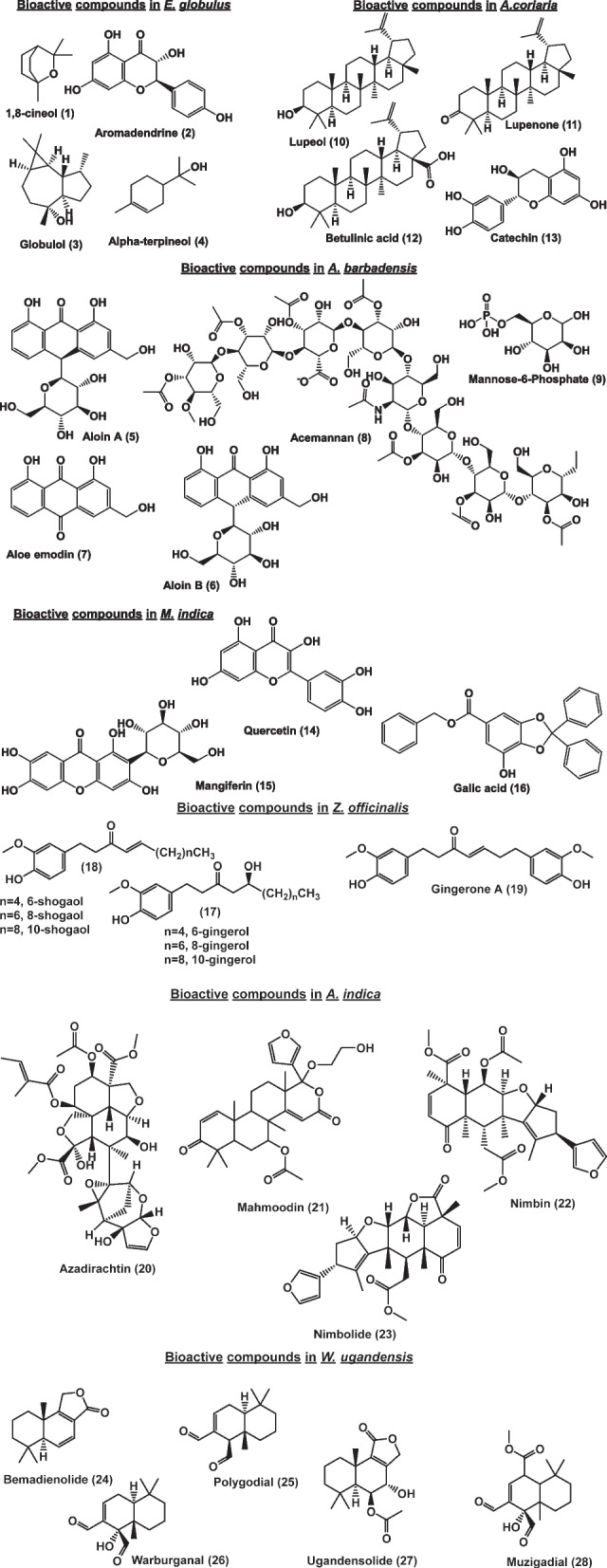
Bioactive compounds in the studied medicinal plants relevant to industrial application (the structures were generated with ChemDraw® software)

For most plant materials, there are compounds with sufficient biological evidence relevant to the industrial application of the products in which the medicinal plant is contained. However, most of the compounds in *Warburgia ugandensis* have not been individually evaluated. The most important bioactive compounds are shown in Fig. 4: *E. globulus (*1,8-cineol (1), aromadendrin (2), globulol (3) and α-terpineol (4)), A. barbadensis; (aloin A (5), aloin B (6), aloe emodin (7), acemannan (8) and mannose 6 phosphate (9)), *A. coriaria*; (lupeol (10), lupenone (11), betulinic acid (12), catechin (13)), M. indica; (catechin (13), quercetin (14), mangiferin (15) and gallic acid (16)), Azadirachta indica; (azadirachtin (20), mahmoodin (21), nimbin (22), and nimbolide (23), Zingiber officinalis; (gingerols (17), shogaols (18) and zingerone (19)), Warburgia ugandensis; (bemadienolide (24), muzigadial, polygodial (25), warbuganal (26), ugandensolide (27), and muzigadial (28).

The biological activities of these compounds and their mechanisms of action are summarized in Table 2 below.

**Table 2:** Evidence of biological activity of the compounds found in the plant materials

Plant species and manufacturer therapeutic claim	Bioactive compound	Evidence of Biological activity of bioactive compound related to traditional use of the plant	Mechanism of action
*Eucalyptus globulus* pain relief, URT disorders, mouth wash, GIT disorders	Aromadendrine (dihydrokaempferol)	Anti-inflammatory Ani-oxidant	Scavenging of reactive oxygen species, chelation of metal ions [16]
		Antibacterial; against *Staphylococcus aureus, Streptococcus mutans*	Inhibits biofilm formation [17]
	1,8-cineol	Antibacterial-against *K. pneumoniae*, *S. aureus*, *P. aeruginosa*	Disruption of bacterial cell membrane & loss of intracellular materials [18]
		Antiasthma, anti-bronchitis, anti COPD, Anti-influenza	Downregulation of inflammation cytokines such as interleukin- 1b (IL-1b) and tumor necrosis factor-a resulting in bronchial muscle relaxant and reduction in mucus secretion [19]
		Anti-inflammatory Ani-oxidant	Regulates nuclear factor-kappa B (NF- κB) and nuclear factor erythroid-2-related factor 2 (Nrf2) pathways [20, 21]
		Analgesic/sedative	Suppression on the CNS by modulating glutamatergic & dopaminergic systems, activates transient receptor potential melastatin 8 [22, 23]
		Antispasmodic and antisecretory, gastroprotective, antidiarrheal	Promotes regeneration of the gastric cells, increases gastric mucus, antioxidant anti-inflammatory effects [20]
	globulol	Antibacterial-against *K. pneumoniae*, *S. aureus*, *P. aeruginosa* [24, 25]	
	α-terpineol	Anti-inflammatory & antioxidant mainly analgesic	Suppresses superoxide production by monocytes; inhibits release of inflammatory mediators including serotonin, histamine, bradykinin, & prostaglandins [26, 27]
		Antibacterial-against *K. pneumoniae*, *S. aureus*, *P. aeruginosa*	As 1,8-cineole
		Anti-gastric ulcer	As 1,8-cineole
*Aloe barbadensis* cracked lips, mouth wash, URT disorders, GIT disorders	Aloin A and B Aloe emodin	Laxative	Inhibition of Na+/K + pump & Cl-channels increase gastric motility; stimulate secretion of mucus and chloride ions [28, 29]
		immuno-modulatory effects	Inhibition of histamine release from mast cells -reduced production of Tumor Necrosis Factor (TNF)-α [30]
		anti-inflammatory- antioxidant	Cyclooxygenase pathways and reducing prostaglandin E2 production [31, 32]
		antibacterial activity-H pylori	Activation of phagocytic leukocytes; inhibition of the N-acetyltransferase activity of *H. pylori* [33, 34]
	Acemannan	Wound healing anti-inflammatory	Activates macrophages to release fibrogenic cytokines; inhibits thromboxane A_2_ [35, 36] Cyclooxygenase pathways and reducing prostaglandin E2 production [30]
	Mannose 6 phosphate	Wound healing	Enhances activity of fibroblasts [37, 38]
	Lupeol	Anti-inflammatory- antioxidant Wound healing effect	Lupeol reduces TNF-α, IL-1, and IL-6 cytokine production. This lowers the infiltration macrophage to damaged tissues, hence reducing inflammation. [39]. It also chelates toxins such as heavy metal ions [40] Stimulates the production, and migration of keratinocytes and fibroblasts to injured tissues, by activating the PI3K-PKB/Akt and p38/ERK/MAPK pathways [41].
*Albizia coriaria* URT disorders,	Lupeol and lupenone	Anti-inflammatory Analgesic	Decreases PGE_2_, TNF-α, interleukin-1b production [42, 43]
		Immunomodulating; anti allergen, anti-asthmatic,	Reduces the production eosinophils, thus interleukins-reduced fluid production in the bronchoalveolar pathway [44]
		Antibacterial-against *S. aureus*, *K. pneumoniae*, *P. aeruginosa* [45, 46]	
		Anti-viral-herpes simplex [47]	Inhibits virus plaque formation [48]
	Betulinic acid/betulin	Anti-inflammatory Analgesic	Inhibits production of nitric oxide & PG_2_ (cyclooxygenase-2 activity); also decreases production of pro-inflammatory cytokines including IL-1β, IL-6, IL-8, IL-12, & TNF [49, 50]
		Antibacterial-*P. aeruginosa*, *S. aureus*, *Mycobacterium tuberculosis* [51]	Enhance the rate of electron transport chain activity, which results in excess production of ROS, which damage DNA, and cause bacterial death [52].
		Anti-viral-against herpes simplex [53]	Inhibits viral plaque formation [48]
	(+/-) Catechin	Anti-inflammatory	Radical scavenging; activates production of erythroid-derived factor 2 which regulates antioxidant enzymes [54]
		Immunomodulatory; anti-allergenic	Reduces production & infiltration of lung tissue by inflammatory cytokines such as TNF-α, IL-1β [55]
		Anti-viral-against Influenza A, SARS-CoV-2 [56, 57]	Inhibits viral receptor binding [58]
*Mangifera indica* URT disorders,	Mangiferin	Antioxidant, anti‑inflammatory, antipyretic, analgesic,	Scavenging of ROS, chelation of toxic metal ions; downregulates phosphorylation of NF- κB pathways-reduces production of proinflammatory cells [59, 60]
		Antiallergic, anti-asthmatic, immunomodulatory	Reduces tracheal contraction by inhibiting the nitric oxide‑cyclic GMP pathway [61]; Inhibits production of nitric oxide and PG2 (cyclooxygenase-2 activity); also decreases production of pro-inflammatory cytokines including [62]
		Antibacterial-activity against *S. aureus* [63]	Increased antibody titers; increases cell mediated immunity. Stimulates lysozyme activity, [64]
		Antiviral-against Herpes simplex [65]	Inhibits viral replication [66]
	(+/-) Catechin, epicatechin	Anti-viral, anti-inflammatory and anti-allergenic	Activity against influenza A and B; catechins inhibit receptor binding and sialidase activities [58]. Catechins regulate the production of proinflammatory agents such as TNF-α, NF-κB, COX-2 in lung tissue. They also scavenge noxious metal ions and ROS [67, 68].
	Gallic acid	Antioxidant, anti-inflammatory	Scavenging of ROS, chelation of toxic metal ions; downregulates phosphorylation of NF- κB pathways-reduces production of proinflammatory cells [50–52]
		Antimicrobial- *P. aeruginosa, S. aureus*	Interferes with colonization by inhibiting motility & adherence; disrupts cell membrane leading to leakage of cell nutrients; inhibits dihydrofolate reductase, topoisomerase IV [69, 70]
		Antiviral - *Haemophilus* influenza A & B	Disruption of the viral particles [71]
	Quercetin	Antioxidant, anti-inflammatory,	Scavenging of ROS, chelation of toxic metal ions; downregulates phosphorylation of NF- κB pathways-reduces production of proinflammatory cells, inhibits cyclooxygenase & lipoxygenase enzymes [67, 72–74]
		Immune-modulatory; anti-allergic	Inhibits IL 8 & 6, TNF-α [75]
		Antibacterial; *P. aeruginosa*, *S. aureus*, *B. subtilis*, *M. tuberculosis*, *K. pneumoniae*	Inhibits nucleic acid synthesis, disrupts plasma membrane, inhibits glutamine synthetase, inhibits biofilm formation [76, 77]
		Antiviral - *Influenza-A* virus [78]	Interacts with Hemagglutinin (HA) glycoprotein to prevent entry into the host cell, thereby inhibiting viral replication. It also inhibits the M2 protein and neuraminidase (NA) glycoprotein interfering with packaging of genome segments into influenza virus particles [79].
*Azadirachta indica* URT disorders, cracked lips	Tetranortriterpenes- Azadirachtin	Anti-inflammatory, antipyretic, antioxidant Wound healing, Anti-gastric ulcer	Inhibits cyclooxygenase (COX), & lipoxygenase (LOX) enzymes. Modulates transcription factors NF-κB, radical scavenging
		Immunostimulant	Inhibits TNF-induced biological responses [80]
		Antibacterial- against *S. aureus* & MRSA	Inhibits biofilm formation [81]
	Nimbidin, Nimbin,	Antipyretic, anti-inflammatory and antioxidant [82, 83]	Suppresses production of inflammatory cytokines from neutrophils & macrophages [84]
		Anti-gastric ulcer	Reduces secretion of gastric acid by inhibiting histamine (H2) receptors and muscarinic receptors [85]
		Immunomodulatory; anti-allergic	Inhibits macrophage migration [86]
	Nimbolide	Antibacterial [87]	
	Mahmoodin	Anti-inflammatory, Antibacterial [88]	
	Diterpens-Margolone, margolonone, margolonone	Antibacterial-against *Klebsiella, Staphylococcus* & *Serratia* Species [88]	
*Zingiber officinalis* URT disorders, GIT disorders	Gingerols 8- gingerol 10-gingerol 12-gingerol 6-gingerol Shogaols 6-shogaol	Antioxidant activity, Anti-inflammatory, analgesic: 6-gnigerol and 6-shogaol most studied	Scavenging of ROS, chelation of metal ions; oxygenation arachidonic acid, a substrate for cyclooxygenase enzymes, thus inhibiting production of prostaglandins; reduced activation of macrophages; inhibit nitrite oxide (NO) production [89, 90]
		Anti-asthmatic, anti-allergen	Reduced contraction of smooth respiratory muscles by reduction in Ca2 + influx & β2 receptor activation; reduced production of proinflammatory cytokines [91]
		Antibacterial- *S. aureus, Mycobacteria, Streptococcus pyogenes*, *Streptococcus pneumoniae, Haemophilus influenzae*	Inhibition of biofilm formation, inhibition of hydroxymethyl-7, 8- dihydro pterin pyrophosphokinase, 6-gingero > 8- gingerol > 10-gingerol > 12-gingerol [92, 93]
		Anti-gastric-ulcer activities -anti- *H. pylori*, 10-gingero > 8- gingerol > 6-gingerol > 6-shogaol [94] -anti-emetic-	Inhibit 5-hydroxytryptamine to increase gastric motility and emptying [95, 96]
	Zingerone (major pungent compound in ginger)	Anti-inflammatory antioxidant activity	Reducing ROS production; chelation of metal ions [97]
		Antibacterial activity	Dihydro pterin pyrophosphokinase inhibition
*Warburgia ugandensis* URT disorders,	Drimane sesquiterpenes Muzigadial, Muzigadiolide Warburganal, warburgadione, warburgadial, warburgin Ugandensidial, Ugandensolide polygodial	Anti-inflammatory and anti-allergic – polygodial	inhibition of phospholipase A2 and neuropeptide release [98]
		Antimicrobial-antimycobacterial (muzigadial & muzigadiolide) [99]	
		Antibacterial (against K. pneumoniae, P. aeruginosa, S. aureus (warburganal, ugandensidial, and polygodial) [100]	

*URT *Upper respiratory tract, *GIT *Gastrointestinal, *ROS *Reactive oxygen species

### Availability of analytical standards and assay methods

For *Eucalyptus globulus*, *Aloe barbadensis*, *Zingiber officinalis*, and *Azadirachta indica*, monographs with well-elaborated quality control methods have been published. In addition, analytical standards for the selected compounds are available, and their assay methods have been developed. On the other hand, no monographs for *Albizia coriaria*, *Mangifera indica*, and *Warburgia ugandensis* exist; for *W. ugandensis*, there are no analytical standards or assay methods to quantify the individual compounds. The cost per mg or mL of analytical standard ranged from €0.6 to 498 for α-terpineol and azadirachtin, respectively, with an average cost of €62.5 ± 101.5. The results are summarized in Table 3.

**Table 3:** Available analytical standards and assay methods for selected medicinal plant compounds

Plant material	Identified Analytical markers	Commercial sources	Smallest commercial unit available, purity and/cost (Euros-€)	Cost per mg/mL of marker (Euros-€))	Analytical methods for the plant material mentioned in local or WHO Pharmacopoeia	Assay methods already developed for the compounds in same or other materials
*Eucalyptus globulus* oil	Aromadendrine (dihydrokaempferol)	Sigma‒Aldrich/Supelco	Analytical standard-530/5 mg ≥95% HPLC grade-221/10 mg ≥ 95% LC/MS-ELSD-421/1 mg	106.0 22.1 421	WHO monographs &African pharmacopoeia-identification tests for 1,8-cineole in oil & leaf materials. TLC fingerprint for leaf materials with 1,8-cineole as reference [10, 13].	Gas chromatography FID & MS method for quantification of oil components [25, 101]
	α-terpineol	Sigma‒Aldrich/Supelco	Analytical standard (≥ 95%)-63.9/100 mg	0.6		
	globulol	Sigma‒Aldrich	≥ 98.5% (sum of enantiomers, GC)- 247/100 mg	2.5		
	1,8-cineol (eucalyptol)	Sigma‒Aldrich/Supelco	Analytical standard- 48.3/1 mL	48.3		
Aloes -whole leaf products	Aloin A	Sigma‒Aldrich	Analytical standard- 440/10 mg	44.0	WHO monographs-Thin-layer chromatography, microchemical analyses to identify anthracenes, Spectrophotometry-to determine total glycosides as aloin [10]	HPLC-DAD/MS quantification methods for anthracenes in aloe vera samples [102]
	Aloin B	Sigma‒Aldrich	Phyproof® Reference Substance- 605/10 mg	60.5		
	Aloe emodin	Sigma‒Aldrich	Analytical standard 311/10 mg	31.1		
*Aloe vera* gel	Acemannan	Toronto Research chemicals	Technical grade- 208.3/10 mg	20.8	Chemical test for carbohydrates; Polysaccharide composition analysis by gas–liquid chromatography [10]	Molecular Exclusion Chromatography [103] UV‒Vis Spectrophotometry [104]
	D-Mannose 6 phosphate	Sigma‒Aldrich	≥ 98% (HPLC) (sodium salt)- 354.0/100 mg	3.5		None found
*Albizia coriaria* stem bark	Lupeol	Sigma‒Aldrich	Analytical standard-130/10 mg	13.0	No monographs for *Albizia coriaria* stem bark materials	HPLC-DAD quantification method for triterpenoids [105, 106]
	lupenone	Toronto Research chemicals	Analytical standard-170/2.5 mg	68.0		HPLC-DAD quantification method for triterpenoids in *Albizia inundata* [107]
	Betulinic acid	Sigma‒Aldrich/Supelco	Analytical standard 95.1/10 mg ≥ 98% (HPLC)- 70/5 mg	9.5 14.0		HPLC-DAD quantification method for betulinic acid in *Albizia lebbeck* [108]
	Catechin	Sigma‒Aldrich/Supelco	Analytical standard − 307/10 mg	30.7		HPLC-DAD quantification method *Albizia lebbeck* [109],
*Mangifera indica* leaf or stem bark	Mangiferin	Sigma‒Aldrich/Supelco	Analytical standard-108/10 mg	10.8	No monographs for *Mangifera indica* leaf or stem bark materials	HPLC-UV quantification method for mangiferin [110]
	Catechin	Sigma‒Aldrich/Supelco	Analytical standard − 307/10 mg	30.7		HPLC-UV quantification method for phenolic compounds [111] HPLC methods for quantification of epicatechin [112, 113]
	Epicatechin	Not found	N/A			
	Gallic acid	Sigma‒Aldrich	Phyproof® Reference Substance − 345/100 mg	34.5		
	Quercetin	Sigma‒Aldrich	United States Pharmacopeia (USP) Reference Standard- 357/200 mg phyproof® Reference Substance- 253/20 mg ≥ 95% (HPLC)- 68.3/10 g	1.8 12.7 6.8		
*Azadirachta indica* stem bark or leaf	Azadirachtin	Sigma‒Aldrich	Phyproof® Reference Substance- 403/5 mg ~ 95%-249/0.5 mg	80.6 498.0	WHO monographs-high-performance liquid chromatography quantification of tetranortriterpenes in oil and leaf materials [11]	HPLC-UV quantification method for Azadirachtins [114]
	Nimbin	Toronto Research chemicals	Analytical standard-105/1 mg	105.0		HPLC-UV quantification method for nimbin [115]
	Nimbidin	Not found	N/A			None found
	Nimbolide	Sigma‒Aldrich	≥ 98%-624/5 mg	124.8		HPLC-UV quantification method for nimbolide [116]
	Mahmoodin	Not found	N/A			None found
*Zingiber officinalis* rhizome	8-gingerol	Sigma‒Aldrich/Supelco	Analytical standard 489.0/10 mg	48.9	WHO monographs-Thin-layer chromatography fingerprinting to identify gingerols and shogaols; quantitative gas chromatography and high-performance liquid chromatography analyses of ginger oils for gingerols, Shogaols [10]	HPLC‒MS quantification methods for gingerols and related compounds [117] [118, 119]
	10-gingerol	Sigma‒Aldrich	Analytical standard-546/10 mg ≥ 98% (HPLC)-276/5 mg phyproof® Reference Substance-472/10 mg	54.6 55.2 47.2		
	12-gingerol	Not found	N/A			
	6-gingerol	Sigma‒Aldrich	Analytical standard- 448/10 mg ≥ 98% (HPLC)- 358/5 mg phyproof® Reference Substance- 472/10 mg	44.8 71.6 47.2		
	6-shogaol	Sigma‒Aldrich	Analytical standard-538/10 mg Phyproof® Reference Substance- 657/10 mg	53.8 65.7		
	Zingerone	Sigma‒Aldrich	Analytical standard- 84.50/50 mg	1.7		None found
*Warburghia ugandensis* stem bark	Polygodial	Sigma‒Aldrich	≥97% (HPLC)-216/10 mg	21.6	No monograph for *Warburghia ugandensis* stem bark	None found
	Bemadienolide	Not available	N/A			None found
	Muzigadial	Not available	N/A			None found
	Warbuganal	Not available	N/A			None found
	Warbugadione	Not available	N/A			None found
	Warbugadial	Not available	N/A			None found
	Warbugin	Not available	N/A			None found
	Ugandensolide	Not available	N/A			None found
	Ugandensidial	Not available	N/A			None found

N/A Not applicable

### Selection of markers

Most of the compounds scored at least 5 points out of 8 except those for *Warburgia ugandensis*, which scored only one point. The scores of the markers for each plant are summarized in Table 4.

**Table 4:** Ranking of the biomarkers

Plant material and manufacturer therapeutic claim	Bioactive Compound	Evidence of Biological activity of bioactive compound	Level of evidence of biological activity related to traditional use of the material	Relative Concentration in plant or extract	Mean Concentration above (5 ppm) %ppm	Mean concentration above 50ppm Yes (1)/no (0) E	Reference Standard available Yes (1)/no (0) F	Analytical methods available? Yes (1)/no (0)	Herb MaRS score
Score		**Yes-1** **No-0** **(A)**	**1 symptom-1** **2 symptoms-2** **3 symptoms-3** **(B)**		**Yes (1)** **No (0)** **(C)**	**Yes (1)** **No (0)** **(D)**	**Yes (1)** **No (0)** **(E)**	**Yes (1)** **No (0)** **(F)**	**/8** **(A + B + C + D + E + F)**
*Eucalyptus globulus* leaf/oil	aromadendrine	1	2	10–30% of essential oil [25]	1	1	1	1	7
	α-terpineol	1	3	0.50% of essential oil [25]	1	1	1	1	8
	Globulol	1	1	10–11% of essential oil [25]	1	1	1	1	6
	1,8-cineol	1	3	97.32% of essential oil [120]	1	1	1	1	8
*Aloe barbadensis* whole leaf/gel	Aloin A and B	1	3	0.1–0.6% of leaf, 10–30% of latex [121, 122]	1	1	1	1	8
	Aloe emodin	1	2	0.09–0.29 mg/g of whole leaf [123]	1	1	1	1	7
	Acemannan	1	2	109-135ppm of gel [103]	1	1	1	1	8
	Mannose 6 phosphate	1	1	ND	0	0	1	0	3
*Albizia coriaria* stem bark	Lupeol	1	3	1–6 mg/g of stembark [106]	1	1	1	1	8
	Lupenone	1	1	19–200 ppm of stem bark [107]	1	1	1	1	6
	Betulinic acid	1	3	1.2–10 mg/g of stem bark [106, 108]	1	1	1	1	8
	Catechin	1	3	0.2–12 mg/g of stem bark [109]	1	1	1	1	8
*Mangifera indica* leaves/stem bark	Mangiferin	1	3	5–20 mg/g of leaves [110]	1	1	1	1	8
	Catechin	1	3	71.4 mg/g of stem bark [111]	1	1	1	1	8
	Epicatechin	1	1	8.07 mg/g of stem bark [111]	1	1	0	1	5
	Gallic acid	1	3	2.08 mg/g of stem bark [111]	1	1	1	1	8
	Quercetin	1	3	0.76 to 1.16 mg/g of leaves [124]	1	1	1	1	8
*Azadirachta indica* leaves/stem bark/seeds	Azadirachtin	1	3	3.8 to 4.8 mg/g of seeds [125] 0.1-1 mg/g of leaves [126]	1	1	1	1	8
	Nimbin,	1	3	0.018 to 0.64 mg/g of oil [115]	1	1	1	1	8
	Nimbolide	1	2	0.9–6.7 mg/g of leaf [116]	1	1	1	1	7
	Mahmoodin	1	2	Not determined	1	1	0	0	5
*Zingiber officinalis* rhizome	12-gingerol	1	2	0.01–0.02 mg/g of rhizome [119]	1	1	0	1	6
	10-gingerol	1	2	0.2–0.4 mg/g of rhizome [117]	1	1	1	1	7
	8-gingerol	1	1	0.4–0.5 mg/g of rhizome [117]	1	1	1	1	6
	6-gingerol	1	3	1.1-2.0 mg/g of rhizome [117]	1	1	1	1	8
	6-shogaol	1	3	0.01–0.02 mg/g of rhizome [119]	1	1	1	1	8
	Zingerone	1	2	ND	0	0	1	1	5
*Warburgia ugandensis* stem bark	Bemadienolide	1	0	ND	0	0	0	0	1
	Muzigadial,	1	1	ND	0	0	0	0	2
	Polygodial	1	2	ND	0	0	1	0	3
	Warbuganal,	1	1	ND	0	0	0	0	2
	warbugadione,	1	0	ND	0	0	0	0	1
	warbugadial,	1	0	ND	0	0	0	0	1
	Warbugin	1	0	ND	0	0	0	0	1
	Ugandensidial,	1	1	ND	0	0	0	0	2
	Ugandensolide	1	0	ND	0	0	0	0	1

Key: *ND *Not determined, *ppm* parts per million

## Discussion

Standardization is a key step in the quality assurance of herbal materials and their products; it is essential to ensure reproducibility of the biological activity and quality of the product. In this study, we established a list of compounds that can be used as markers for seven of the most commonly used plant materials in Uganda. Our emphasis was on compounds that are known to be active such that their determination informs both the quality and efficacy of the materials. We hope this information will be relevant to manufacturers once the new National Drug Authority (NDA) guidelines are put in force; quantification of markers and establishment of chromatographic fingerprints will be needed (https://www.nda.or.ug/wp-content/uploads/2022/03/Guidelines-on-Regulation-of-Traditional-and-Local-Herbal-Medicnes-in-Uganda_Draft-2.pdf).

With the use of a modified herbal marker ranking system, compounds were identified that can be utilized to control the quality of herbal materials. Evidence of biological activity, availability of the analytical standard and availability of an analytical method are paramount. Thus, a compound should score at least 5 points to be suitable, that is, 3 points for biological activity and one point each for analytical standard and analytical method availability. The minimum concentrations of the markers in the plant material, if not already known, can be established by the manufacturer, and the compound assigned a qualitative (≤ 50 ppm) or quantitative (≥ 50 ppm) role depending on the concentrations in the plant material [5]. Another important factor to consider in quantitative analysis is the cost of the marker compounds. In this study, we highlight unit costs and the costs of the smallest packs for each compound. It is important to note, however, that the final acquisition costs will include vendor or agent markups and so might be significantly higher. While 1 mg or 1 mL is considered sufficient for external calibration, other forms of calibration, such as standard addition, will require higher amounts of the marker. To show the relevance of the selected markers, we list situations for which standardization of each plant material is needed.

### Markers for *Eucalyptus globulus* oil

*Eucalyptus globulus* is known for the essential oils obtained from the leaves of the plant. The oil is used to manufacture products such as syrups used to manage symptoms of respiratory tract disorders (cough, common cold, catarrh, sore throat, congestion from asthma, bronchitis, allergic conditions- sinusitis, rhinitis, hiccups), fever and measles; pain balms applied topically to manage pain and inflammation; and mouth washes for conditions such as toothache, bad odor, sensitive teeth, bleeding gums, tooth cavities, tooth decay, and mouth sores [7]. Some of the most studied compounds that exhibit pharmacological activities to support the indications include aromadendrine, α-terpineol, globulol, and 1,8-cineol (Table 2; Fig. 4), with scores of 7, 8, 6, and 8, respectively. Since all the compounds are available in pure form and several quantitative methods have been established (Table 3), these compounds are all suitable as markers. Aromadendrine is the most expensive, with a unit cost of €183), while α-terpineol is the cheapest, with a unit cost of €0.6. The WHO and the African pharmacopoeia recommend the use of 1,8-cineol as a standard for both chemical reaction and TLC fingerprint identification methods [10, 13]; however, some studies have shown aromadendrine to be the major component, and perhaps a multimarker approach is more appropriate than determining only cineol [25]. These markers can be used to authenticate, determine phyto-equivalence and monitor the consistency in quality of oils obtained from different subspecies and geographical locations.

### Markers for *Aloe vera (Aloe barbadensis)*

*Aloe vera* is used as the gel, latex or whole leaf extract. Whole leaf and latex products are used to treat constipation, to “cleanse” the GIT, and to treat wounds [7]. The main active ingredients are anthraquinone glycosides, notably aloin (barbaloin A and B) and aloe emodin [33] (Fig. 4). The efficacy of these compounds is well established, their analytical standards are available, and many analytical methods have been published (Tables 2 and 3). Therefore, aloin A and B scored 8 points, while aloe emodin scored 7 points. The unit cost of analytical standards for aloin A is €44.0, that of aloin B is €60.5, and that of aloe emodin is €31.1. The WHO monograph recommends chemical and TLC methods for the identification of anthracenes and spectrophotometric determination of total anthracene glycosides as barbaloin equivalents for quantitative analysis [10]. These markers can be employed in identifying aloes obtained from different geographical regions, determining the best geographical sources of aloe vera gel products [123], and standardizing aloe products marketed for the treatment of constipation [10].

Because of suspected carcinogenicity [127], some regulatory authorities have banned the inclusion of aloe (whole leaf and latex) in oral over-the-counter nutraceuticals and laxative products. For instance, the International Aloe Science Council set a limit of 10 ppm total anthraquinone glycoside concentration (as aloin), while the European Medicines agency and Food and Drug authority set the limit at 0 ppm [128, 129]. In this case, aloin and aloe emodin are negative markers and can be used to assess the quality of over-the-counter products. However, the Uganda National Drug Authority and Uganda Bureau of Standards have not published any regulations on the use of aloes.

The main components of the gel are carbohydrates such as glucomannans and sugars [103]). One of the main compounds, acemannan, scored 8 points. Its biological activity is well studied, analytical markers are available and analytical methods have been developed (Tables 2 and 3). The main sugar, mannose 6 phosphate, scored only 3 points since its bioactivity is not well studied, and there are no analytical methods; thus, its concentration in the gel has not been reported. The unit cost of acemannan is approximately €20.8. For quality assurance of *Aloe vera* gel, the WHO monograph recommends a chemical test for carbohydrate and polysaccharide analysis by GC/MS. A molecular exclusion chromatographic method and a UV‒Vis spectrophotometric method for polysaccharides have also been published and are more affordable.

Assay methods for acemannan can be used to select high-yielding plant varieties, the best cultivation sites, suitable agronomic practices, and harvest seasons [130, 131].

### Markers for *Albizia coriaria*

The dried stem bark is the plant material of interest for medicinal purposes. Commercial products are used for managing symptoms of respiratory tract disorders (whooping cough, catarrh, sore throat, congestion from asthma bronchitis, fever, sinusitis) [7]. Some pharmacologically active compounds, such as triterpenoids, lupeol, lupenone, betulinic acid and betulin [132, 133] (Fig. 4), have been elucidated with scores of 8, 6, 8, and 8, respectively. These compounds possess biological activities relevant to the commercial uses of the products, are available in pure form and have been quantified in many Albizia species, although assay methods specific to *A. coriaria* are scarce. The unit costs range between €68 for betulinic acid and €9.5 for betulinic acid.

There are no pharmacopoeial methods or monographs for *A. coriaria* materials. The identified markers can be used to monitor the batch-to-batch consistency of raw materials [134, 135] and to evaluate the efficiency of extraction methods.

### Markers for *Mangifera indica*

The pharmacologically active compounds are obtained from extracts of the stem bark and leaves of *Mangifera indica*. The products containing these extracts are used for the management of respiratory tract disorders (whooping cough, catarrh, sore throat, congestion from asthma and bronchitis) [7]. Several phenolic compounds have been characterized and shown to possess biological activity relevant to the medicinal use of the materials. Mangiferin, catechin, quercetin and gallic acid scored 8. The unit costs of these markers ranged from €1.8 for quercetin to €34.5 for gallic acid. While epicatechin scored 5 points, its analytical standard is not readily available.

The identified markers can be used to monitor the batch-to-batch consistency of raw materials, to select the most suitable plant cultivars to source from [111] and to control the extraction and processing methods. There are no monographs for *M. indica* materials [7].

### Markers for *Azadirachta indica*

The seed oil, leaves and stem bark are used as herbal materials. Products containing these herbal materials are used to manage respiratory tract disorders (cough, flu, sore throat, sinusitis), gastrointestinal disorders (gastric ulcers, flatulence, constipation) and lip balms (dry, cracked, and painful lips) [7]. The most important compounds are the limonoid azadirachtin and the tetranortriterpenes [84] nimbin, nimbidin, nimbolide and mahmoodin (Fig. 4). Most compounds scored 7 and above and are therefore suitable markers. The unit costs of these markers range from €105 to 289 for nimbin and azadirachtin, respectively. Although nimbolide and mahmoodin scored 5 points, they lack analytical standards and assay methods. According to the WHO monographs, high-performance liquid chromatography quantification of oxidized tetranortriterpenes in oil and leaf materials can be used for quality control [11]. Assays of these markers can be applied to select habitats for cultivation of neem, determine the best harvesting season and ensure consistency of materials obtained from various sources [115, 136, 137].

### Markers for *Zingiber officinalis *(Ginger)

Herbal material is obtained from the rhizome, and the products are used to manage symptoms of respiratory tract disorders (cough, flu, sore throat, sinusitis, bronchial asthma, and fever) [7]. Gingerols and their dehydration products, shogaols (Fig. 4), have been extensively studied [89]. The compounds possess several pharmacological activities relevant to the application of the products; their analytical standards are readily available (apart from 12-gingerol), and analytical methods have been published. Thus, all compounds scored between 6 and 8 points and are thus suitable as markers. The unit cost of the analytical standards ranges between 50 and 60 euros. The WHO monographs recommend TLC fingerprinting with gingerols and shogaols as standards and GC and HPLC assay methods [10]. Since gingerols are dehydrated to form shogaols during storage and upon exposure to heat [138], the ratio of gingerols to shogaols can be used to determine the freshness of the ginger samples and optimize storage conditions. The quantities of the markers can be applied to optimize extraction processes and to study the phyto-equivalence of gingers obtained from different sources [139].

### Markers for *Warburgia ugandensis*

The bark of the stem is used as a drug for the treatment of respiratory tract disorders (cough, measles symptoms, common colds, sinusitis, rhinitis, asthma, catarrh, bronchial congestion, and hiccups) and gastric ulcers [7]. While many compounds have been elucidated, the most important being the drimane sesquiterpenes bemadienolide, muzigadial, polygodial, warburganal, ugandensolide, and muzigadial [99] (Fig. 4), specific bioactivity studies are rare.

Only polygodial, muzigadial muzigadiolide, warburganal, and ugandensidial have been shown to possess some antimycobacterial activity [99]. In addition to limited pharmacological evidence, most of these compounds are not available in pure form for use as analytical standards, and no assay methods have been published. Thus, the compounds scored between only 1 and 3 points and are therefore not suitable quality control markers according to the Herb MaRS. In such cases, the WHO recommends the use of other constituents, whose biological activities are known even though the relevance of such activities to the traditional use of the plant may not be well established [3]. Thus, compounds such as linoleic acid, myrcene, and linalool, which are known components of W. ugandensis [140], can be used for its quality control; such evaluation will not be relevant to pharmacological standardization of the plant materials. This lack of pharmacological data, analytical methods and standards is common to plants that are exclusively used in Africa [8].

## Conclusions

This study has demonstrated the applicability of the Herb MaRS to the quality assurance of herbal materials. Markers have been identified for the phytochemical standardization of the six most common medicinal plants in Uganda. The selected markers were as follows: (aromadendrine, α-terpineol, globulol, and 1,8-cineol) (in *Eucalyptus globulus* Labill. ); (aloin, aloe emodin, acemannan) (in *Aloe barbadensis* (L.) Burm.f. ), (lupeol, lupenone, betulinic acid, betulin, and catechin) (in *Albizia coriaria* Oliv.); (mangiferin, catechin, quercetin, and gallic acid (in *Mangifera indica* L.); (azadirachtin, nimbin, nimbidin (in *Azadirachta indica* A.Juss. ); and (6,8,10-gingerols, and 6-shogaol (in *Zingiber officinalis* Roscoe). For *W. ugandensis*, the compounds with known biological activity were not suitable as markers because they lack analytical standards and/or analytical methods. This implies that the Herb MaRS is only applicable for plants that have been extensively researched, such that it is possible to establish evidence of efficacy and/or safety. The method is also only applicable to plants whose phytochemical ingredients have analytical standards and corresponding analytical methods.

### Recommendations

Markers for the other twenty-five (25) plant materials should be established using the same approach. The identified markers should be evaluated for suitability at the various stages of the production chain of herbal medicines in Uganda, i.e., from authentication and quality control of raw materials to evaluating reproducibility in the efficacy, safety, and stability of finished products notified by the National Drug Authority. Information about marker evaluation can be included in future Ugandan medicinal plant monographs and/or product databases to guide their quality assurance. In addition to the quantification of marker compounds, the construction of fingerprint databases for various plants is encouraged. The standardized fingerprints can then be used for routine quality assessment of the plant materials.

## Data Availability

The datasets during and/or analyzed during the current study are available from the corresponding author upon reasonable request.
